# Intention to seek professional psychological help among college students in Turkey: influence of help-seeking attitudes

**DOI:** 10.1186/1756-0500-6-519

**Published:** 2013-12-06

**Authors:** Feyza Seyfi, Krishna C Poudel, Junko Yasuoka, Keiko Otsuka, Masamine Jimba

**Affiliations:** 1 Department of Community and Global Health, Graduate School of Medicine, The University of Tokyo, 7-3-1, Hongo, Bunkyo-ku, Tokyo 113-0033, Japan; 2 Department of Public Health, School of Public Health and Health Sciences, University of Massachusetts Amherst, 316 Arnold House, 715 North Pleasant St, Amherst, MA 01003-9304, USA

**Keywords:** Professional psychological help, Attitudes, Intention, Help seeking, College students, Mental health, Turkey

## Abstract

**Background:**

Depression rates are high among college students in Turkey, but often students do not seek mental health care. This study aimed to examine the association between attitudes toward seeking professional psychological help and intention to seek professional psychological help among such college students. We also examined the factors associated with students’ professional psychological help-seeking attitudes. We conducted this cross-sectional study among 456 conveniently sampled graduate and undergraduate students in Ankara. We collected students’ data using self-administered, structured questionnaires in the Turkish language and then analyzed the data using both descriptive and multivariate methods.

**Results:**

In the multiple linear regression analyses, students’ attitudes towards seeking professional psychological help were positively associated with intention to seek such help (p < 0.001). Other factors positively associated with students’ attitudes towards seeking professional psychological help included the following: age (p < 0.001), perceived social support from family (p < 0.05), perceived social support from friends (p < 0.01), and perceived social support from significant other (p < 0.05). Students with less positive attitudes toward seeking professional psychological help were more likely to be men (p < 0.001), undergraduate students (p < 0.001), and students who were not aware of the presence of the on-campus counseling center (p < 0.01).

**Conclusions:**

Students’ positive attitudes toward seeking professional psychological help were positively associated with their intentions to seek such help. To encourage utilization of the counseling center inside the campus more frequently when in need, interventions might be necessary to improve students’ attitudes toward seeking professional psychological help—in particular among young male students.

## Background

Mental disorders constitute about 14% of the global burden of diseases. Such high morbidity is caused by conditions including depression, psychoses, anxiety, and other mental disorders [1]. According to the World Health Organization (WHO), 31.7% of all disability-adjusted life-years (DALYs) are due to mental disorders with depression as the leading cause (11.8%) [1]. Moreover, depressive disorders are expected to account for the highest proportion of DALYs by 2030 in high-income countries, the second highest in middle-income countries, and the third highest in low-income countries [2].

In Europe, after cardiovascular diseases, mental disorders account for the highest burden of disease [3]. Depression alone is the third greatest cause of all years lived with disability in the region, accounting for 6.2% across different age groups. Young people are also prone to suffer from mental disorders; the reported rates of depression among college students from 11 countries in Europe were around 20% on an average [4].

College students need to cope with the psychological and social changes that accompany the development of an autonomous life during the college years [5]. This is because these changes may lead to mental disorders; disorders at this stage of life can then have a prolonged impact on emotional, social, physical, and cognitive development in this critical period of development [6]. Therefore, students should be encouraged to seek professional psychological help early in the progression of the disease.

Overall, professional psychological help-seeking behavior remains low in the general population in Turkey; the percentage of the people who required mental health care but did not seek professional treatment was estimated to be 60% [7]. Among the population, mental disorders are most prevalent between 15 and 29 years of age [8]. This population segment, which includes college students, has unacceptably high rates of depression and other mental disorders, ranging from 10% to 40% [9,10]. Despite the presence of on-campus counseling services [11] and high rates of depression among college students, 70% of the college students in Turkey do not utilize professional psychological help [12]. Instead, they tend to seek psychological help from informal sources, such as friends and family, when they feel psychologically distressed [13].

Culture influences willingness to seek help [14]. Culture also influences many aspects of mental illness, including how patients from a given culture express and manifest their symptoms, their style of coping, family and community support, and their willingness to seek treatment. Likewise, the cultures of the clinician and the service system influence diagnosis, treatment, and service delivery. Due to its unique location bridging Europe and Asia, Turkey has both Western and Eastern cultural values and both collectivism and individualism [15].

In Turkey, little attention has been given to professional psychological help-seeking behavior. Psychological help-seeking behavior has been defined as a behavior in which a person actively searches for psychological assistance from a mental health provider such as psychiatrists, psychologists, and counselors [16][17]. Attitudes toward seeking professional psychological help have been examined previously in Turkey, and in these studies college students showed unfavorable attitudes toward seeking professional psychological help [18].

According to the theory of reasoned action [19], intention to perform a behavior is a direct predictor of the behavior. As for the prediction of intention, attitudes are the most direct predictors of intentions. Attitudes have been defined as “the degree to which a person has favorable or unfavorable evaluation or appraisal of the behavior in question,” and they have been incorporated into many human social behavior theories such as the health belief model, social cognitive theory, and the theory of planned behavior. More attitudes that are positive lead to greater intentions to perform a behavior [19]; therefore, the people with positive attitudes toward professional psychological help seeking are more likely to seek help than the people with negative attitudes [20]. It is, therefore, important to know more about the factors that influence and shape people’s attitudes toward psychotherapy and their intentions to seek help.

Social support has been known in a few studies to be associated with attitudes toward seeking professional psychological help. In some studies, low social support was associated with more positive attitudes toward seeking professional help [21][22]. Individuals who perceive less social support from people in their social networks might resort to professional help when they experience psychological problems [23]. On the other hand, in other studies, social support was found to be positively associated with attitudes [24]. If a person is encouraged to seek professional help by his or her peers, he or she might develop more positive attitudes toward seeking professional help. In one study in Turkey, women and students who had higher levels of social support from family and friends showed more positive attitudes toward seeking professional help than men and students with lower levels of such social support [13].

According to the literature, gender is another important factor that influences help-seeking behavior. Female gender is associated with more favorable attitudes toward seeking professional psychological help, and consequently, with higher levels of intention [13][25][26].

Therefore, we found it important to add these demographic and psychological variables in our research while examining the relationship between attitudes and intention. Such knowledge could facilitate the development of interventions aimed at increasing favorable attitudes toward psychotherapy, and consequently, could promote service utilization early on rather than during advanced stages of mental disorder.

The current study builds upon previous research in Turkey examining the attitudes toward seeking professional psychological help. We examined the role of previously examined psychological (i.e., perceived social support) and demographic (i.e., prior counseling experience, gender) factors in predicting students’ attitudes toward seeking help. The association between attitudes toward seeking professional psychological help and intention to seek professional psychological help has been studied in the United States [27], Asia [28], Australia [29], and the U.K. [30]. However, no similar study has been conducted in Turkey, despite the high prevalence of mental health conditions among young people [10]. In this study, we first aimed to replicate the previously demonstrated positive association between attitudes toward seeking professional psychological help and intention to seek help. We also aimed to replicate the gender differences in help-seeking attitudes to show that women have more positive attitudes toward help seeking than men. Finally, we explored the effect of perceived social support on help-seeking attitudes as the findings in the literature are contradictory.

## Methods

### Study area and design

We conducted this cross-sectional study in Middle East Technical University (METU) in Turkey. Turkey is a middle-income country in Europe and has a lifetime prevalence of mental disorder of 12%.

The METU is a large state university located in Ankara, the capital city of Turkey. A psychological counseling center is available inside the campus where university students can consult the counselors and psychologists by appointment without any fees involved. Apart from giving personal counseling service, the counseling center frequently organizes two-hour group workshops for students on topics related to coping with depression, anxiety, fears, and improving body image. Counseling center advertises through brochures and posters hung on bulletin boards inside the departments.

### Data collection

We received approval from METU to conduct this study, and we contacted 10 conveniently selected professors. Among them, nine gave their consent to participate in the study. We then recruited students during the lectures of those professors who agreed to assist with data collection. Out of 463 conveniently-selected students, we collected data from 456 graduate and undergraduate students, and 7 students refused to participate in the study. We excluded 20 students from the analyses because of missing data. The selected students were enrolled in the engineering, education, and natural sciences faculties. We collected data using self-administered structured questionnaires in the Turkish language during October 2011. The questionnaire was distributed to students in the classrooms after obtaining permission from the relevant lecturers, under the supervision of the first author.

### Measures

Measures included intention to seek professional psychological help, attitudes toward seeking professional psychological help, and perceived social support. We also collected data on socio-demographic characteristics: age, gender, faculty, current degree program, prior counseling experience for psychological problems, source of psychological help when experiencing psychological problems, and awareness of the on-campus counseling center. Professional psychological help was defined as formal help provided by mental health professionals (e.g., psychologist, psychiatrist, and counselor).

#### Intention to seek professional psychological help

Professional psychological help-seeking intention was assessed using a one-item question, as suggested by Ajzen [31]. Students rated the likelihood of seeking help from a professional in the event that they were to have psychological problems in the future from 1 (*not likely at all*) to 7 (*very likely*).

#### Attitudes toward seeking professional help

Help-seeking attitudes were measured using the Attitudes toward Seeking Psychological Help Scale-Shortened Form (ASPH-S) scale [25], a shortened 18-item revision of the Attitudes toward Seeking Psychological Help Scale (ASPH) [32]. The scale was developed with university students in Turkey in the Turkish language. It utilizes a 5-point Likert-type scale ranging from 1 (*totally disagree*) to 5 (*totally agree*), with 6 items reverse scored. Higher scores reflect more attitudes that are positive. The scale has satisfactory internal consistency (0.88) and test-retest reliability (0.99) [25]. In this study, Cronbach’s alpha for the scale was 0.89.

#### Perceived social support

Perceived social support was measured with the Turkish version of the Multidimensional Scale of Perceived Social Support (MSPSS) [33]. The scale has three factor subscales: family support, friend support, and significant other support. The scale utilizes a 12-item, 7-point Likert-type scale from 1 (*very strongly disagree*) to 7 (*very strongly agree*). Each subscale consists of four items, and higher scores signify more perceived social support. Translation of the scale into Turkish along with the requisite reliability and validity study in Turkey were done by Çakır and Palabıyıkoğlu [34] with a sample of young people (12–22 years old). The psychometric properties of the scale among college students in Turkey was tested by Duru [35], who concluded that the scale can be used for Turkish college students as well. The scale has satisfactory internal consistency (0.76) and test-retest reliability (0.81) [34]. In this study, Cronbach’s alpha was 0.87 for the whole scale, and for family, friend, and significant other subscales, it was 0.90, 0.90, and 0.95, respectively.

#### Socio-demographic characteristics

We collected information on age, gender, current degree program, and faculty. We measured students’ age in years. Gender was a categorical variable with two choices: “male” or “female.” Current degree program was likewise measured as a categorical variable: “undergraduate” or “graduate.” Faculty was an open-ended item, and each student filled in the specific name of his or her affiliated faculty.

#### Prior counseling experience for psychological problems

Prior counseling experience was assessed using a one-item question with two response options: “yes” or “no”. All students were asked whether they had ever consulted a mental health professional for psychological problems regardless of their history of experiencing psychological problems.

#### Sources of psychological help when experiencing psychological problems

In order to assess the choice of psychological help source, the students were asked one question: from whom do they seek help when they experience psychological problems? This question was posed to all the students regardless of their history of experiencing psychological problems. Students could choose from seven response options indicated in the questionnaire: mental health professional, family, friends, significant other, books, the Internet, and no one. More than one answer was possible for this item.

#### Awareness of the on-campus counseling center

Awareness of the on-campus counseling center was assessed with one dichotomous yes/no question. Additionally, we asked those who were aware of the counseling center for the source of this knowledge; the students could choose from six possible means: friends, the Internet, academic supervisor, orientation program, brochure, and posters. More than one answer was possible for this item.

### Data analysis

We performed descriptive analysis using the chi-square tests for categorical variables and t-tests for continuous variables. Subsequently, simultaneous multiple linear regression analyses were run.

First, we used multiple linear regression analysis to examine the roles of attitudes toward seeking professional psychological help in predicting intention to seek professional psychological help. In this analysis, the dependent variable was intention to seek help, and the independent variables were age, gender, faculty, degree program, prior counseling experience, awareness of the on-campus counseling center, perceived social support, and attitudes toward seeking professional psychological help. We also tested for a moderating effect of gender on the relationship between attitudes and intention.

Finally, we ran a multiple linear regression analysis to examine the role of demographic and psychological factors in predicting attitudes toward seeking professional psychological help. In this analysis, the dependent variable was attitudes toward seeking professional psychological help, and the independent variables were age, gender, faculty, degree program, prior counseling experience, awareness of the on-campus counseling center, and perceived social support.

We selected the independent variables in the multiple regression analyses based on the previous studies on professional psychological help-seeking [36][37]. Data were analyzed using Statistical Package for the Social Sciences (SPSS) version 18.0. An alpha level of 0.05 was used for all statistical tests.

### Ethical considerations

Prior to the distribution of the questionnaire, we informed the students about the aim of the study and the content of the questionnaire; the voluntary nature of participation was also conveyed. We obtained verbal consent from all participants and excluded those who refused to participate in the study without any repercussions. The participants did not receive any incentives in return for their participation in the study. We carefully ensured the confidentiality of the participants’ information; we did not collect any names or identifying pieces of information from the students. We obtained ethical approval from the Research Ethics Committee of the Graduate School of Medicine, the University of Tokyo and from the Human Subjects Ethics Committee of the Middle East Technical University, Turkey. Permission was also granted by the relevant authorities and professors at the Middle East Technical University to collect data during their lectures.

## Results

Table 1 shows the descriptive characteristics of the study participants. Around 20% of the students had ever consulted a mental health professional (such as psychologist, psychiatrist, psychological counselor) for psychological problems. Most students turned to their families and friends for psychological help when they experienced problems; only a small number of students sought help from professionals. Half of the students were not aware that there was a counseling center on campus, and those who were aware had acquired this knowledge during the orientation program and through their friends. Brochures and posters distributed by the center could reach only a small percentage of the students.

**Table 1:** Descriptive characteristics of the participants (N = 436)

Variables	N	%	M	SD
Age (years)			22	3
Gender				
Female	237	54.4		
Male	199	45.6		
Degree program				
Undergraduate	251	57.6		
Graduate	185	42.4		
Faculty				
Engineering & Natural Sciences	212	49.6		
Education	224	51.4		
Prior counseling experience*				
Yes	85	19.5		
No	351	80.5		
Main source of psychological help†**				
Mental health professional	54	12.4		
Family	280	64.2		
Friend	303	69.5		
Significant other	124	28.4		
Books	77	17.7		
Internet	87	20.0		
No-one	46	10.6		
Awareness of the on-campus counseling center				
Yes	217	49.8		
No	219	50.2		
Source of this knowledge				
Friend	96	44.7		
Internet	24	11.1		
Academic supervisor	27	12.5		
Orientation program	86	38.9		
Brochure	24	11.1		
Posters	12	5.6		

Note. N = number of participants; % = frequency; M = mean; SD = Standard deviation.

*All participants, regardless of psychological distress, answered this question.

**The sources from whom students seek help when they experience psychological problems.

†More than one answer was possible.

Women were more likely to seek professional psychological help than were men (t = 6.38, p < 0.001). Similarly, women had more positive attitudes toward seeking professional help than did men (t = 6.15, p < 0.001) (Table 2).

**Table 2:** Mean scores on intention, attitudes, and perceived social support scales by gender among students

	**Total (N = 436)**		**Female students (N = 237)**		**Male students (N = 199)**		
**Variables**	**Mean**	**SD**	**Mean**	**SD**	**Mean**	**SD**	**p-value**
Intention*	5.5	1.8	5.9	1.4	4.9	2.0	< 0.001
ASPH-S (Attitudes)	69.7	10.5	72.5	8.7	66.2	11.3	< 0.001
MSPSS (Social support)	63.3	14.7	65.1	14.1	61.3	15.3	0.680
Family support	22.5	5.8	23.3	5.9	21.6	5.7	< 0.001
Friend support	22.8	5.3	23.3	5.0	22.3	5.6	0.027
Significant other support	18.0	9.1	18.5	9.0	17.4	9.3	0.188

Note. ASPH-S = Attitudes toward Seeking Professional Psychological Help Scale Shortened; MSPSS = Multidimensional Scale of Perceived Social Support.

*Intention to seek professional psychological help.

### Factors associated with intention to seek professional psychological help

Table 3 shows the results of the multiple regression analysis for factors associated with intention to seek professional psychological help. Compared to women, men had lower intentions to seek professional psychological help (β = -0.096, p < 0.05). Students with higher levels of positive attitudes toward seeking professional psychological help also had higher intentions to seek professional psychological help (β = 0.605, p < 0.001). Students who did not have prior counseling experience showed lower levels of intentions to seek professional psychological help (β = -0.087, p *<* 0.05). Gender did not moderate the relationship between help-seeking attitudes and intention.

**Table 3:** Simultaneous multiple regression analysis for the prediction of intention to seek professional psychological help

**Variable**	**B**	**SE B**	**β**
Age	-0.028	0.032	-0.049
Gender※	-0.331	0.142	0.093*
Faculty†	0.018	0.031	0.023
Degree program‡	0.055	0.207	0.015
Prior counseling experience¶	-0.402	0.171	-0.090*
Awareness of the on-campus counseling center§	-0.113	0.133	-0.032
Attitudes (ASPH-S)	0.083	0.022	0.493***
Perceived social support (MSPSS)			
Family support	0.007	0.012	0.021
Friends support	0.002	0.014	-0.003
Significant other support	-0.001	0.008	-0.072
Gender X attitudes♣	0.120	0.013	0.113

Note. Overall regression results, F(11,419) = 29.65, p < 0.001, r^2^ = 0.42.

ASPH-S = Attitudes toward Seeking Professional Psychological Help Scale-Shortened;

MSPSS = Multidimensional Scale of Perceived Social Support. ※Reference group = female;

†Reference group = engineering & natural sciences faculty; ‡Reference group = undergraduate;

♣Interaction term testing the moderating effect of gender on the relationship between attitudes and intention.

¶Reference group = Yes; §Reference group = Yes. *p < 0.05 **p <0.01 *** p < 0.001.

### Factors associated with attitudes toward seeking professional psychological help

Table 4 shows the results of the multiple regression analysis for factors associated with attitudes toward seeking professional psychological help. Older students were more likely than their younger counterparts to have positive attitudes toward seeking professional psychological help (β = 0.186, p < 0.01). Compared to women, men had less positive attitudes toward seeking professional psychological help (β = -0.242, p < 0.001). Similarly, graduate students showed less positive attitudes toward seeking professional psychological help (β = -0.225, p < 0.001). Those students who were not aware of the presence of a counseling center on campus also showed less positive attitudes (β = -0.119, p < 0.01). Students who reported higher levels of family support had more positive help-seeking attitudes (β = 0.098, p < 0.05), as did those with higher levels of friend support (β = 0.142, p < 0.01) and higher levels of significant other support (β = 0.094, p < 0.05).

**Table 4:** Simultaneous multiple regression analysis for the prediction of attitudes toward seeking professional psychological help

**Variable**	**B**	**SE B**	**β**
Age	0.618	0.226	0.186**
Gender※	-5.103	0.973	-0.242***
Faculty†	0.416	0.217	0.091
Degree program¶	-4.764	1.434	-0.225***
Prior counseling experience‡	-1.819	1.206	-0.069
Awareness of the on-campus counseling center§	-2.486	0.932	-0.119**
Perceived social support (MSPSS)			
Family support	0.176	0.085	0.098*
Friends support	0.283	0.097	0.142**
Significant other support	0.108	0.054	0.094*

Note. Overall regression results, F(9,421) = 11.45, p < 0.001, r^2^ = 0.18.

MSPSS = Multidimensional Scale of Perceived Social Support.

※Reference group = female; †Reference group = engineering & natural sciences faculties;

¶Reference group = undergraduate; ‡Reference group = Yes; §Reference group = Yes.

*p < 0.05 **p < 0.01 ***p < 0.001.

## Discussion

The goal of this study was to examine the gender differences in attitudes toward seeking professional psychological help and the influence of attitudes on intention to seek help. We found that more positive attitudes toward seeking professional psychological help were positively associated with higher intention to seek such help among college students in Turkey. Moreover, women had more positive attitudes and higher intentions to seek professional psychological help than did men.

Our study indicated that students’ attitudes toward seeking professional psychological help were positively associated with their intention to seek such help. In the USA [21], Asia [28], Australia [29], and Europe [30], those students with more positive attitudes toward professional psychological help seeking were more likely to seek help than those with less positive attitudes. Ajzen [17] expected that the pattern of influence of attitudes on intention should be universal, although there might be variations in the contribution. His assertion is supported by studies conducted in different cultures [38]. Therefore, it is necessary to address students’ attitudes toward counseling in order to reach out to those in need of mental health care services.

Men were less likely to seek professional psychological help than were women. Although our study did not address reasons why men were less likely to seek help, it might be because of traditional gender roles where men are expected to be strong, self-sufficient, and in control in emotional situations [39]. In this context, for men, seeking psychological help may mean admitting to being weak and dependent on other people [40].

Among men, having prior counseling experience was also associated with higher intention to seek professional psychological help. This may be due to a tendency for prior experience to put men at ease, combatting the cultural expectation that men should be autonomous [41].

Younger students were less likely to have positive attitudes toward seeking professional psychological help. This could be because younger students may have difficulties in identifying and describing their emotions [42] and are more self-reliant due to the increasing need for autonomy that comes with transition to college life [43].

Men showed less positive help-seeking attitudes than women, as has been consistently shown [41][26]. This can also be explained by traditional male roles and masculinity ideology, the belief that men should conform to culturally defined male roles (i.e., they should be strong, self-reliant, and independent) [44]. Therefore, men may avoid showing their full range of emotions and try to deal with their problems without outside assistance [45].

In this study, we also found that undergraduate students had more positive attitudes toward seeking professional psychological help than did graduate students. Future qualitative studies are warranted to explore this association between degree program and professional psychological help-seeking attitudes.

Awareness of the on-campus counseling center was another factor associated with more positive attitudes toward seeking professional psychological help. By way of explanation for this phenomenon, students who are aware of the counseling center may also be more knowledgeable of the services, and therefore, may have less stigma against counseling.

Although some studies have shown that less perceived social support was associated with more positive attitudes toward seeking professional psychological help [21][22], in the present study, higher levels of perceived family, friend, and significant other support were associated with more positive attitudes toward seeking professional psychological help. This finding is consistent with previous study conducted in Turkey [13]. In this study, most students reported that they sought psychological help from family members and friends rather than professionals. However, students receiving high levels of social support may interact with their family members, friends and/or significant others more often than students with lower levels of social support, which, in turn, enables them to establish trusted relationships [46]. This interaction may include talking and exchanging ideas; therefore, in the event that a family member, friend, or significant other has positive attitudes toward seeking professional psychological help or has had previous counseling experience, the students might be influenced by them and develop more positive attitudes [47]. Moreover, students with higher social support may develop social skills which enable them to disclose and describe their emotions more easily—a prerequisite for psychological therapies [47].

Results of this study should be interpreted in light of several limitations, which, in turn, provide some direction for future research. First, the cross-sectional nature of the study limits the interpretation of causality, but the data provide observational support that more positive attitudes toward seeking professional psychological help are associated with higher intention to seek such help. Several other longitudinal studies [43][48][49] are also in line with our identification of help-seeking attitudes as a predictor of intention. Second, we recruited students only from one university in Turkey; therefore, the generalizability of the results to all the college students in Turkey is unknown. Third, we assessed the intention to seek professional psychological help and not the actual help-seeking behavior of the students. However, intention has been established as a direct predictor of help-seeking behavior [36]; the correlation between intention to seek professional psychological help and actual help seeking is positive and significant [43]. Fourth, our study used some measurements that are liable to introduce recall bias, which may conceivably result in over- or under-reporting. However, the provision of a comfortable environment in familiar classroom spaces was designed with a view to reduce any such limitation.

Future research should examine actual help-seeking behaviors and other factors explaining the low intentions among the students. Future investigations could also examine the results of educational programs and other interventions targeted toward increasing positive attitudes.

## Conclusions

More positive attitudes toward seeking professional psychological help were associated with higher intentions to seek such help among university students in Turkey. We, therefore, recommend that interventions should improve such attitudes among college students in Turkey to encourage the students to utilize the on-campus counseling center more frequently and consult the psychiatrists, psychologists, or counselors when in need. Such interventions might include screening programs and stigma-reduction campaigns on the campus level. These interventions should particularly target young, male graduate students. In particular, support of family, friends, and significant others might improve attitudes toward seeking professional psychological help.

## Abbreviations

WHO: World health organization; DALY: Disability adjusted life years; METU: Middle East Technical University; ASPH: Attitudes toward seeking psychological help scale; ASPH-S: Attitudes toward seeking psychological help scale-shortened; MSPSS: Multidimensional scale of perceived social support; SPSS: Statistical package for the social sciences.

## Competing interests

Authors declare that they have no competing interests.

## Author’s contributions

FS designed the study, collected data, performed statistical analyses, and wrote the manuscript. KCP participated in the design of the study, provided valuable guidance in the data collection, and was actively involved in statistical analyses and manuscript writing. JY and ON helped to draft and revise the manuscript and provided guidance. MJ monitored the study progress and contributed to the study design and revisions of the article. All authors read and approved the final manuscript.
